# Supplementary material for: School health programs of physical education and/or diet among pupils of primary and secondary school levels I and II linked to body mass index: A systematic review protocol within the project From Science 2 School
Source: PLoS One. 2022 Oct 6;17(10):e0275012. doi: 10.1371/journal.pone.0275012 (PMC9536596; doi:10.1371/journal.pone.0275012)
Supplement: Attachment — Submitted filename: PONE-D-21-25090_reviewer.pdf [file pone.0275012.s002.pdf]

PLOS ONE

School health programs of physical education and/or diet among pupils of primary and secondary school levels I and II in relation to body mass index: a systematic review protocol within the project From Science 2 School

--Manuscript Draft--

Manuscript Number:	PONE-D-21-25090
Article Type:	Study Protocol
Full Title:	School health programs of physical education and/or diet among pupils of primary and secondary school levels I and II in relation to body mass index: a systematic review protocol within the project From Science 2 School
Short Title:	School health programs of physical education and/or diet: a systematic review protocol
Corresponding Author:	Derrick Tanous<br>University of Innsbruck: Universitat Innsbruck<br>Innsbruck, AUSTRIA
Keywords:	body mass index; body weight; School children; Physical education; Diet
Abstract:	Introduction : t he most common causes of death in Western countries today are preventable diseases mainly attributed to daily behavior. It has been well documented that genetics are influential but not the deciding factor for develop ing non-communicable disease s . Ideally, the public should be educated to perform methods of optimal health and wellbeing independently, meaning that individuals should be in control of their health without relying on others. As behavior is known to be consistent over time, good or poor health behavior will track from childhood in to adul thood. Physical activity and diet are permanently linked to the individual's state of health , and when properly balanced, the effects on personal health summate, resulting in greater benefits from this dual-approach for public health. Objective : to highlight the different approaches (physical intervention, nutritional intervention, and dual-approach of diet and exercise) and identify effective intervention s for sustainable body weight and healthy body mass index in school children. Method s: A systematic review will be conducted following the Preferred Reporting Items for Systematic Review and Meta-Analysis (PRISMA) guidelines. The review will assess school-based diet and exercise interventions on children in primary and secondary school levels I and II. Discussion: Overweight and o besity develop as a result of a prolonged imbalance in the energy balance model, with both physical activity and diet being influential in the fluctuation of body weight. A dual-approach including physical activity and diet could therefore be a very promising method to promote sustainable healthy body weight in school children.
Order of Authors:	Derrick Tanous
	Gerhard Ruedl
	Werner Kirschner
	Drenowatz Clemens
	Joel Craddock
	Thomas Rosemann
	Katharina Wirnitzer
Additional Information:	
**Question**	**Response**
**Financial Disclosure**<br><br>Enter a financial disclosure statement that describes the sources of funding for the work included in this submission. Review	This work was supported by the doctoral scholarship University of Innsbruck Vice Rectorate for Research for promoting young scientists (Author DT): No. 2020/2/PSY/SPORT-21. URL: https://www.uibk.ac.at/ffq/forschungsfoerderung/2020/dok-stips-20-3.tranche/ausschreibung.html

Powered by Editorial Manager® and ProduXion Manager® from Aries Systems Corporation

the submission guidelines for detailed requirements. View published research articles from *PLOS ONE* for specific examples.<br><br>This statement is required for submission and **will appear in the published article** if the submission is accepted. Please make sure it is accurate.<br><br>**Unfunded studies**<br>Enter: *The author(s) received no specific funding for this work.*<br><br>**Funded studies**<br>Enter a statement with the following details:<br>• Initials of the authors who received each award<br>• Grant numbers awarded to each author<br>• The full name of each funder<br>• URL of each funder website<br>• Have or will the sponsors or funders play any role in the study design, data collection (when applicable) and analysis, decision to publish, or preparation of the manuscript?<br>• **NO** - Include this sentence at the end of your statement: *The funders had and will not have a role in study design, data collection and analysis, decision to publish, or preparation of the manuscript.*<br>• **YES** - Specify the role(s) played.<br><br>* typeset	The funders had no role in study design, data collection and analysis, decision to publish, or preparation of the manuscript.
**Competing Interests**<br><br>Use the instructions below to enter a competing interest statement for this submission. On behalf of all authors, disclose any competing interests that could be perceived to bias this work—acknowledging all financial support and any other relevant financial or non-financial competing interests.<br><br>This statement is required for submission and **will appear in the published article** if the submission is accepted. Please make sure it is accurate and that any funding sources listed in your Funding Information later in the submission form are also	The authors have declared no competing interests exist.

declared in your Financial Disclosure statement.<br><br>View published research articles from *PLOS ONE* for specific examples.<br><br>**NO authors have competing interests**<br><br>Enter: *The authors have declared that no competing interests exist.*<br><br>**Authors with competing interests**<br><br>Enter competing interest details beginning with this statement:<br><br>*I have read the journal's policy and the authors of this manuscript have the following competing interests: [insert competing interests here]*<br><br>* typeset	
**Ethics Statement**<br><br>Enter an ethics statement for this submission. This statement is required if the study involved:<br><br>• Human participants<br>• Human specimens or tissue<br>• Vertebrate animals or cephalopods<br>• Vertebrate embryos or tissues<br>• Field research<br><br>Write "N/A" if the submission does not require an ethics statement.<br><br>General guidance is provided below. Consult the submission guidelines for detailed instructions. **Make sure that all information entered here is included in the Methods section of the manuscript.**	N/A

Powered by Editorial Manager® and ProduXion Manager® from Aries Systems Corporation

**Format for specific study types**<br><br>**Human Subject Research (involving human participants and/or tissue)**<br>• Give the name of the institutional review board or ethics committee that approved the study<br>• Include the approval number and/or a statement indicating approval of this research<br>• Indicate the form of consent obtained (written/oral) or the reason that consent was not obtained (e.g. the data were analyzed anonymously)<br><br>**Animal Research (involving vertebrate animals, embryos or tissues)**<br>• Provide the name of the Institutional Animal Care and Use Committee (IACUC) or other relevant ethics board that reviewed the study protocol, and indicate whether they approved this research or granted a formal waiver of ethical approval<br>• Include an approval number if one was obtained<br>• If the study involved *non-human primates*, add *additional details* about animal welfare and steps taken to ameliorate suffering<br>• If anesthesia, euthanasia, or any kind of animal sacrifice is part of the study, include briefly which substances and/or methods were applied<br><br>**Field Research**<br><br>Include the following details if this study involves the collection of plant, animal, or other materials from a natural setting:<br>• Field permit number<br>• Name of the institution or relevant body that granted permission	
**Data Availability**<br><br>Authors are required to make the data underlying their research findings fully available, without restriction.<br><br>If the manuscript reports pilot or preliminary results, the data underlying those results must be made available at the time of publication. A Data Availability	N/A - the protocol does not report results

Powered by Editorial Manager® and ProduXion Manager® from Aries Systems Corporation

Statement describing where the data can be found is required at submission. Your answers to this question constitute the Data Availability Statement and will be published in the article, if accepted. When legal or ethical requirements prohibit public data sharing, authors must indicate how researchers may obtain access to the data. See the PLOS Data Policy and FAQ for detailed information.<br><br>**Important:** Stating 'data available on request from the author' is not sufficient. If your data are only available upon request, select 'No' for the first question and explain your situation in the text box.<br><br>For protocols without pilot or preliminary data, authors are strongly encouraged to state how they plan to share research data from their study when it is completed or published.<br><br>Do the authors confirm that EITHER the protocol does not report results OR all data underlying the preliminary results will be made fully available without restriction?	
**Describe where the data may be found in full sentences. If you are copying our sample text, replace any instances of XXX with the appropriate details.**<br><br>• If **no pilot data are reported** in the manuscript, please enter the following: *No datasets were generated or analysed during the current study. All relevant data from this study will be made available upon study completion.*<br>• If the manuscript reports pilot data **held in a public repository**, include URLs, accession numbers or DOIs. If this information will only be available after acceptance, indicate this by ticking the box below. For example: *All XXX files are available from the XXX database*	No datasets were generated or analysed during the current study. All relevant data from this study will be made available upon study completion.

(accession number(s) XXX, XXX.).<br>• If the manuscript reports pilot data contained **within the manuscript and/or Supporting Information files**, enter the following:<br>*All relevant data are within the manuscript and its Supporting Information files.*<br>• If neither of these applies but you are able to provide **details of access elsewhere**, with or without limitations, please do so. For example:<br><br>*Data cannot be shared publicly because of [XXX]. Data are available from the XXX Institutional Data Access / Ethics Committee (contact via XXX) for researchers who meet the criteria for access to confidential data.*<br><br>*The data underlying the results presented in the study are available from (include the name of the third party and contact information or URL).*<br>• This text is appropriate if the data are owned by a third party and authors do not have permission to share the data.<br><br>For study protocols with data management or sharing plans, authors are encouraged to briefly describe in their Data Availability Statement how data they generate will be made accessible when the study is completed. For example:<br>• Deidentified research data will be made publicly available when the study is completed and published.<br><br>* typeset	
Additional data availability information:	

Powered by Editorial Manager® and ProduXion Manager® from Aries Systems Corporation

Manuscript

Click here to access/download;Manuscript;PLOS ONE_School Health Programs_Tanous_3Aug2021.docx

School health programs of physical education and/or diet among pupils of primary and secondary school levels I and II in relation to body mass index: a systematic review protocol within the project *From Science 2 School*

Tanous Derrick R[1], Ruedl Gerhard[1], Kirschner Werner[1], Drenowatz Clemens[2], Craddock Joel[3] Rosemann Thomas[4], Wirnitzer Katharina[1,5,6,7]

Author Affiliations

[1]Department of Sports Science, Leopold-Franzens University of Innsbruck, Austria 185 Fürstenweg, 6020 Innsbruck Austria; [2]Division of Physical Education, University of Education Upper Austria, Linz, Austria; [3]Sydney School of Education and Social Work, The University of Sydney, Sydney 2006, Australia.; [4]Institute of Primary Care, University of Zurich, Zurich, Switzerland; [5]Department of Research and Development in Teacher Education, University College of Teacher Education Tyrol, Innsbruck, Austria; [6]Health and Lifestyle Science Cluster Tirol, Subcluster Health/Medicine/Psychology, Tyrolean University Conference, Verbund West, Innsbruck, Austria; [7]Research Center Medical Humanities, Leopold-Franzens University of Innsbruck, Innsbruck, Austria

*Corresponding author: Derrick R Tanous derrick.tanous@student.uibk.ac.at

Email:
Gerhard Ruedl gerhard.ruedl@uibk.ac.at
Werner Kirschner werner.kirschner@uibk.ac.at
Clemens Drenowatz clemens.drenowatz@ph-ooe.at
Joel Craddock joel.craddock@sydney.edu.au
Thomas Rosemann thomas.rosemann@usz.ch
Katharina Wirnitzer katharina.wirnitzer@ph-tirol.ac.at

Abstract

Introduction**:** the most common causes of death in Western countries today are preventable diseases mainly attributed to daily behavior. It has been well documented that genetics are influential but not the deciding factor for developing non-communicable diseases. Ideally, the public should be educated to perform methods of optimal health and wellbeing independently, meaning that individuals should be in control of their health without relying on others. As behavior is known to be consistent over time, good or poor health behavior will track from childhood into adulthood. Physical activity and diet are permanently linked to the individual's state of health, and when properly balanced, the effects on personal health summate, resulting in greater benefits from this dual-approach for public health. ***Objective*****:** to highlight the different approaches (physical intervention, nutritional intervention, and dual-approach of diet and exercise) and identify effective interventions for sustainable body weight and healthy body mass index in school children. ***Method*****s:** A systematic review will be conducted following the Preferred Reporting Items for Systematic Review and Meta-Analysis (PRISMA) guidelines. The review will assess school-based diet and exercise interventions on children in primary and secondary school levels I and II. ***Discussion:*** Overweight and obesity develop as a result of a prolonged imbalance in the energy balance model, with both physical activity and diet being influential in the fluctuation of body weight. A dual-approach including physical activity and diet could therefore be a very promising method to promote sustainable healthy body weight in school children.

Key words: Body mass index; Body weight; School children; Physical education; Diet

Introduction

The most common causes of death in Western countries are non-communicable diseases (NCDs), which can be mainly attributed to daily lifestyle behaviors [1-11]. Up to 71% of the world's deaths per year (41 million) are caused by NCDs [12]. Developing the symptoms of NCDs is a slow process that occurs over decades and results in pain and suffering in the long-term for affected individuals and their families, with nine out of the ten leading causes for years lived with disability attributed to NCDs [13]. NCDs are widely known to be preventable, even at a low cost [1-3, 6, 7, 9, 12, 171]. It has been well documented that genetics are influential but not the deciding factor for developing chronic (non-communicable) diseases [2, 4, 5, 8, 14-18].

Given the importance of behavior in preventing NCDs, health promotion through proper education on developing and maintaining personal health and wellbeing may be a feasible solution to improve the burden of NCDs [19, 20, 171, 174]. Nevertheless, personal health behavior is just one of the determinants of health in addition to genetics, social circumstances, health care, and environmental factors [21, 22].

To maximize personal health care, the public could be educated through competence-orientated health literacy to learn how to control one's health independently before relying on others, such as commercial suppliers [23]. Two key environments where children grow up and develop their health behaviors are: (1) at home and (2) at school [24]. Schools provide a viable intervention setting, as they allow a large number and variety of children and adolescents to be reached independently of their socio-economic background [4, 8, 10, 19, 25-30]. School settings differ from clinical settings and are well controlled in terms of age groups (school levels), state educational mandate of national curricula, and standardized teacher education at tertiary level (such as University level, specialized University College of Teacher Education). Due to their educational efforts, schools influence lifestyle choices and contribute to developing lifelong health promotion and disease prevention [4, 8, 10, 19, 25-32].

Children and adolescents who are overweight or obese have an increased risk for developing chronic diseases over the lifespan [7, 9, 21, 33-36, 171, 173]. Ruedl et al. 2018 concluded that “evidence-based preventative measures to decelerate the rise in body mass index (BMI) of primary school children should be implemented at the earliest” [37]. Overweight/obesity BMI classification is an indicator for excess body fat suggested by the World Health Organization and Centers for Disease Control and Prevention to classify children and adolescents (underweight, normal, overweight, obese 1-3) [36, 38-40]. In addition to biological and genetic aspects, the BMI category is likely related to the individual’s physical activity (PA) level and diet [36, 38, 39, 41]. According to the energy balance model, excess body fat results from consuming more calories per day than calories burned [42]. Therefore, increasing daily PA levels or making adjustments to diet composition can result in slowing, stopping, or reversing the accumulation of excess body fat [42].

There is strong scientific evidence of the beneficial effects of PA regarding the prevention of chronic disease [1, 3, 4, 6-10, 17, 18, 43-59]. Regular PA or physical exercise is not only the key to achieving optimal health but is considered as “medicine,” which no pill or supplement can replace [1, 3, 4, 6-10, 17, 18, 43-58]. Diet is another fundamental pillar in the development of optimal health and wellness [2, 5, 60-94], which can also function as a “medicine” for health but only if the diet is well-planned and balanced, containing mostly plant-based, whole foods [2, 5, 60-65, 67-82, 84-90, 95-98]. The Academy of Nutrition and Dietetics (AND) stated in their position paper that appropriately planned vegetarian, including vegan, diets are healthful, nutritionally adequate, and may provide health benefits for the prevention and treatment of certain diseases and that these diets are appropriate for all stages of the life cycle (pregnancy, infancy, childhood, adulthood, and old age as well as for athletes) [82]. Moreover, studies have consistently shown that people eating plant-based (vegetarian, vegan) diets have healthier BMI on average compared to people eating a mixed (omnivorous) diet [62-64, 67, 99, 100].

PA interventions, dietary interventions, and the dual-approach (permanent linkage or combination of PA and diet) have been implemented in the school setting to improve BMI and body weight (BW), as children and adolescents spend a great deal of time at school [4, 10, 17, 25, 37, 43, 44, 101-107, 166].

Although PA is known as an effective tool for improving personal fitness and shaping good health, PA interventions in schools have been shown to be insufficient to reverse overweight/obesity in the majority of pupils with the condition [44, 101, 103, 108, 166]. Participation in the compulsory subject of physical education (PE) at school is a well-studied and well-functioning opportunity to begin developing the behaviors that lead to a lifetime of PA [4, 43, 44, 109]. However, PA is just one pillar of health, while diet displays another important pillar of health [1, 3, 6, 7, 9, 10, 23, 77, 110-115, 168]. Moreover, school-based dietary interventions have also been shown to be inconsistent in reversing overweight/obesity in most pupils with the condition [10, 26-29, 108].

Considering overweight and obesity develops from an imbalance between energy intake and expenditure, it would be unlikely to stop, prevent, or reverse the condition by solely focusing on PA or diet [42]. The most promising approach appears to be an interaction of PA and diet as an effective solution for sustaining ideal BW [10, 26-29, 166-169, 171, 172]. PA permanently combined with diet is consistently linked to the individuals' state of health, and when properly balanced, the effects on personal health summate, resulting in superior benefits from a "dual-approach" [2, 10, 26-29, 44, 60-62, 64, 116, 166, 167, 171, 172]. Achieving sustainable health in children and adolescents, at best, would target overweight/obesity with lifestyle factors that appear every day, naturally [23]. Lifestyle factors, such as physical activity level and diet, can improve health immediately for benefits that also carry over into adulthood and older adulthood and could pass on to following generations as well [23, 174]. Considering the cumulative benefits to overall health, a dual-approach of PA with a plant-based diet appears most promising [1, 3, 6, 7, 9, 10, 23, 26-29, 36, 110-115, 117].

A large number of school-based interventions have targeted health behaviors, including diet and/or PA [10, 26-29, 118-132, 166]. Based on our cumulative expertise, a variety of research on school-based PA interventions or dietary interventions exists. However, from the authors´ experience, the focus of plant-based dietary intervention is expected to be low. Future school-based health interventions should therefore consider investigating the PA and plant-based diet dual-approach. On an international scale,

there has yet to be a compilation of interventions on PA, diet/diet type, and both for comparing the most effective strategies to improve BMI and/or BW in school pupils. This review aims to determine the best practice of PA, dietary, or combined PA and dietary intervention in primary and secondary school pupils for improving BMI and/or BW. This review aims to answer the following questions:

(1) Is compulsory physical education (PE) as part of the curriculum associated with BMI in school pupils?

(2) What is the minimum duration for compulsory PE to cause a change in BW or BMI in school pupils?

(3) Is compulsory PE with additional PA, sports, or exercise intervention in the school setting associated with healthy BMI in pupils?

(4) Are there differences in the efficacy of school-based physical exercise intervention versus diet intervention in promoting a healthy BMI in pupils?

   a) Subgroup analysis: which kinds of dietary interventions* (Omnivorous: animal-products; Whole Food Plant-Based: fruits, vegetables, legumes, and/or whole grains; Other: not related to diet type e.g. soft drink) implemented in school programs are associated with healthy BMI in pupils?

   b) Subgroup analysis: which interventions (PA, diet, or dual-approach) are associated with healthy sustainable BW management in the long term (one year or more)?

   c) Subgroup analysis: as a dual-approach, are there differences in the efficacy of specific diet scheme types* (omnivore vs. vegetarian vs. vegan) linked to PE for maintaining healthy BW?

*Diet type definitions based on AND 2016 [82].

Methods

Inclusion criteria (2.1)

Types of Studies (2.1.1)

We will include all types of randomized controlled trials (RCTs), including factorial, cluster, crossover, and parallel designs. As school settings clearly differ from clinical settings, we will also include non-randomized trials if any of the research questions cannot be addressed by available randomized controlled trials [133]. The following types of non-randomized trials will be included, if necessary: quasi-randomized controlled trials, controlled before-after studies, and follow-up studies such as inception cohort studies and non-randomized controlled trials.

Types of Participants (2.1.2)

This review will consider all articles on human pupils aged 5 – 19 years only in primary school and secondary school levels I and II (or equivalent), with systematic review parts 1 (secondary schools) and 2 (primary schools) separated by school level. The subjects must have a stable or compensated medical condition without physical or cognitive disability (e.g., cannot follow all the amount and magnitude of lessons and exercises planned in PE lessons) and no prescribed medication. Unless results are separated, the following studies will be excluded as we cannot guarantee a bias-free result for these subjects (as they would likely add extreme values to the data): (i) studies addressing both pupils and adults (ii) studies with a whole school approach based on the inclusion aspect of people with disabilities unable to fully participate.

Types of Intervention (2.1.3)

Interventions of focus must be only school-based and include those related to:

(1) PA – possible variations include:

   (a) PE – compulsory lessons as part of the curriculum (state mandate).

(b) PA – body movements produced by skeletal muscles that result in energy expenditure, not related to PE (e.g., brain/active break: allows bouts of in-class PA without education, or physically active lessons during the learning task at hand, unrelated to the lesson but occurs simultaneously) [134].

(c) Physical exercise – planned, structured, and repetitive sessions with a final or intermediate objective to improve or maintain physical fitness (physical fitness is a set of attributes that are either health- or skill-related) [134].

(d) Sports – related to the development of humans that require physical effort, skills (development of human capacities), a contest including a contract that is rule-governed, institutionalized, and parties have shared values and interests.

(e) Various combinations of (a) – (d).

(2) Dietary: aimed at regulating certain nutrition-related activities or actions that have an impact on food choices and health outcomes, which are not related to increasing BMI in anorexic or underweight youth (e.g. canteen-based, dietary supplement, soft drink reduction).

(3) Dual-approach: combination of PA and dietary as one intervention.

Types of Outcomes (2.1.4)

The main outcomes of interest include BMI and BW and must be calculated or measured by the testers, not self-reported. BMI is defined as body mass (BW) in kilograms divided by height in meters squared (kg/m^2), or by using imperial units with the equivalent calculation. BMI percentile or Z-score will be accepted if BMI (kg/m^2) is not available.

The priority of outcomes includes BMI and BW because these measures are typically assessed in school health-related interventions, as they are cost-efficient, fast, and non-invasive. Moreover, BMI is likely related to the individuals' PA level and diet, and BW is necessary to control for BMI.

Timing (2.1.5)

No restriction on years considered.

Language (2.1.6)

We will include articles reported in English or German language.

Exclusion criteria (2.1.7)

- Classes with an extraordinary pedagogical approach focusing on the needs of one or two pupils.
- Specialized school branches targeting physical exercise, sports in general, or a specific discipline of sport.
- Intervention groups targeting only overweight and/or obese pupils.
- Interventions outside of regular school hours.
- No comparator/no control group.
- No outcome on BMI or BW.

Information sources (2.2)

The following databases will be searched for articles published in English/German: PubMed, EMBASE, Education Source.

Planned dates of completing the search include from October 1 - October 31, 2020.

To aid in the integrity of the search coverage, reference lists of included studies will be scanned as well as relevant reviews identified by the search. A bibliography of included articles will be sent to each member of the review team. PROSPERO (International Prospective Register of Systematic Reviews: https://www.crd.york.ac.uk/PROSPERO/) will be searched to identify similar reviews in progress as well as recently completed reviews.

Search Strategy (2.3)

Only quantitative, published studies will be sought. There will be no study design, date, or language restrictions included as part of the search. Although the databases may vary in the definition of a text word or standardized subject terms search (e.g., Medical Subject Headings [MeSH]), each database search will follow as closely as possible to that of the given PubMed Advanced search in Table 1. To ensure the most accurate coverage of EMBASE, truncation will be used on singular key terms to remove all additional endings (e.g., plural forms) within the search (e.g., Pupil* instead of pupil OR pupils). The only limit used will be to exclude conference abstracts from the final hits of EMBASE. The specific search strategy will be created by the team, which includes expertise in sport science, nutrition science, and specialized statisticians, and peer-reviewed by the University of Innsbruck Library to assist in the structure of the search. Five columns: population 1, population 2, intervention, outcome, and study design will be used to include all related articles. The following steps will be used to search each database:

(1) all terms will be searched independently (e.g., “boy”) and combined with “or” for each column to create five separate search strings

(2) the columns will be combined into one search with “and” linking the five columns (all terms from Population 1 in one string “and” all terms from Population 2 in one string “and” all terms from Intervention in one string “and” all terms from Outcome in one string “and” all terms from Study design in one string).

Table 1: Key Terms for PubMed Advanced Search

The text word [tw] search is used for each term independently unless explicitly given as a MeSH term.

"Or" is included between each row "and" is included between each column during the search.

Population 1	Population 2	Intervention	Outcome	Study Design
1. Pupil	1. First grade	1. Physical intervention	1. Body weight [MeSH:NoExp]	1. Randomized controlled trial
2. Pupils	2. Second grade	2. Physical activity	2. Body weight	2. Randomised controlled trial
3. Boy	3. Third grade	3. Physical activities	3. Body mass	3. Randomized Controlled Trial [Publication Type:NoExp]
4. Boys	4. Fourth grade	4. Sport	4. Body mass index	4. Randomized control trial
5. Girl	5. Fifth grade	5. Sports	5. Body mass index [MeSH]	5. Randomised control trial
6. Girls	6. Sixth grade	6. Run	6. BMI	6. RCT
7. Children	7. Seventh grade	7. Running	7. Body weight management	7. Factorial
8. School children	8. Eighth grade	8. Weight lifting		8. Cluster
9. School kids	9. Ninth grade	9. Progressive strength training		9. Crossover
10. Kid	10. Tenth grade	10. Progressive resistance training		10. Parallel
11. Kids	11. Eleventh grade	11. Weight exercise		11. Intervention
12. Adolescent	12. Twelfth grade	12. Weight training		12. Quasi randomized controlled trial
13. Adolescents	13. Junior high	13. Power exercise		13. Quasi randomised controlled trial
14. Teen	14. High school	14. Power training		14. Cohort
15. Teens	15. High schools	15. Strengthening exercise		15. Cohort study
16. Teenager	16. School	16. Strength exercise		
17. Teenagers	17. Schools	17. Strength training		
18. First graders	18. Primary school	18. Resistive exercise		
19. Second graders	19. Primary schools	19. Resistance exercise		
20. Third graders	20. Elementary school	20. Resistive training		
21. Fourth graders	21. Elementary schools	21. Resistance training		
22. Fifth graders	22. Secondary school	22. Balance training		
23. Sixth graders	23. Secondary schools	23. Core training		
24. Seventh graders	24. Middle school	24. High intensity interval training		
25. Eighth graders	25. Middle schools	25. High intensity training		
26. Ninth graders		26. High intensity exercise		
27. Tenth graders		27. Walking training		
28. Eleventh graders		28. Walking		
29. Twelfth graders		29. Aerobic training		
30. Freshmen		30. Aerobic exercise		
31. Sophomore		31. Anaerobic training		
32. Sophomores		32. Anaerobic exercise		
33. Young adult		33. Endurance exercise		

34. Young adults<br>35. Middle schoolers<br>36. High schoolers<br>37. Youth		34. Endurance training<br>35. Fitness training<br>36. Fitness exercise<br>37. Fitness exercises<br>38. Physical training<br>39. Physical exercise<br>40. Physical exercises<br>41. Low intensity training<br>42. Low intensity exercise<br>43. Light training<br>44. Light exercise<br>45. Active breaks<br>46. Active classroom<br>47. Active school<br>48. Active schools<br>49. Diet<br>50. Diets<br>51. Diet type<br>52. Diet types<br>53. Nutrition<br>54. Nutritional<br>55. Weight loss<br>56. Weight loss program<br>57. Weight loss intervention<br>58. Diet program<br>59. Diet intervention<br>60. Nutrition Program<br>61. Nutritional Program<br>62. Nutrition intervention<br>63. Nutritional intervention		

Study Records (2.4)

All retrieved articles from each database will be transferred to Covidence (a systematic review management site: covidence.org), and all hits will be shared with all authors. On Covidence, title/abstract and full-text screening will be performed by the two reviewers working independently with the protocol. Covidence will remove most duplicates of the same report. If there are any conflicts, the reviewers will solve these by discussion using an online audio application. If the conflict cannot be solved, a third reviewer will help make the final decision. Following the title/abstract screening, an included article will move to a full-text screening by two reviewers working independently for agreement with the eligibility criteria.

Assessment will be made of the author's names, institutions, journal of publication, and results, and multiple reports of the same study will be linked. Contact with authors will be made by email or phone, if necessary, to identify missing information or clarifications for eligibility. Reasons for exclusion will be noted by both reviewers.

Data collection process (2.5)

Two reviewers will collect data extraction through standardized electronic data forms within Covidence (online) platform, and the results of the articles will be collected in MS Office Excel (Version 16.0.14131.20278). The second reviewer will check the outcome extracted to be sure of no missing information or errors in the outcome data collected.

The reviewers are both content area experts, and disagreements will be handled by a third reviewer. If important information cannot be found within the text, the study authors will be contacted by email or phone. The following data items will be extracted:

Table 2. Data Extraction Items

Source:<br>• Study ID (if available)<br>• Citation and contact details	Intervention:<br>• Total number of intervention groups<br>*For each intervention and comparison group of interest*:<br>○ Specific intervention<br>○ Type/regimen of physical activity and/or dietary intervention:<br>○ Intervention details (duration, volume, intensity – sufficient for replication, if feasible).<br>○ Integrity of intervention. (may not be reported)
Eligibility:<br>• Confirm eligibility for review<br>• Reason for exclusion	Outcomes:<br>• Outcomes and time points (i) collected; (ii) reported*.<br>*For each outcome of interest*:<br>○ Outcome definition (with diagnostic criteria, if relevant)<br>○ Unit of measurement (if relevant).<br>○ or scales: upper and lower limits, and whether high or low score is good.
Methods:<br>• Study design<br>• Total study duration<br>• Sequence generation<br>• Allocation sequence concealment<br>• Blinding<br>• Other concerns about bias	Results:<br>• Number of participants allocated to each intervention group.<br>*For each outcome of interest*:<br>○ Sample size<br>○ Missing participants<br>○ Summary data for each intervention group (e.g. 2×2 table for dichotomous data; means and SDs for continuous data).<br>○ Subgroup analyses. (may not be reported)
Participants:<br>• Total number<br>• Setting/school type<br>• Diagnostic criteria<br>• Age<br>• Sex<br>• Country (location if available – urban vs. rural)	Miscellaneous:<br>• Funding source<br>• Key conclusions of the study authors<br>• Miscellaneous comments from the study authors<br>• References to other relevant studies<br>• Correspondence required<br>• Miscellaneous comments by the review authors
Comparator:<br>• Performance of only dietary or physical activity intervention, or control group with detailed description	

Risk of Bias (2.6)

If necessary to include non-randomized study designs, the ROBINS-I tool will be used to assess the risk of bias in non-randomized studies as well as quasi-randomized trials and all of the included domains will be assessed for bias with no additional domains [136, 137]. The bias domains include pre-intervention confounding bias, pre-intervention selection bias, at-intervention information bias, post-intervention confounding bias, post-intervention selection bias, post-intervention information bias, and post-intervention reporting bias [137].

Possible confounding domains include age, sex, BMI pre-intervention, BW pre-intervention, school level, school type, school policies on PA and diet, socioeconomic status, school environment, home environment, active transport, food availability, or baseline PA and dietary pattern.
Possible co-interventions include PA education sessions (without performing PA), health counseling unrelated to PA or diet (e.g. drug awareness), cognitive training, or other youth clubs unrelated to PA or diet (e.g. religious groups).

The Cochrane tool as a part of Covidence will be used to assess the risk of bias in randomized studies [138]. Two reviewers will assess included studies independently based on the following:

- Quality of allocation sequence generation
- Quality of treatment allocation concealment from study participants, clinicians, and other health care personnel from enrollment
- Appropriate blinding of the intervention allocation for team members assessing outcomes and data analysis during the trial
- Quality in the completeness of outcome data addressed in the published report for participant exclusions, attrition, and incomplete outcome data

- Quality of outcome reporting and if there exists evidence of selective outcome reporting which may have affected the study results
- Other possible trial problems that could cause a high risk of bias

We will include a description of the procedure for each domain of bias assessment for every study, including quotes when possible. For each domain, the judgment of the bias will be ranked as “high risk”, “low risk”, or “unclear”. Disagreements will be settled by discussion using an online audio application and, if necessary, a third reviewer. The reviewers will not be blinded to the studies, and agreement between reviewers will not be evaluated.

Data Synthesis (2.7)

Data will be synthesized separately for randomized and non-randomized studies (if included). A descriptive summary of included studies will be incorporated into tables based upon the population (average age of participants), intervention (PA, dietary, combination), comparator (type of diet, active control, passive control), outcome (BW, BMI), and study design (PICOS structure). To answer our research questions and sub-analyses, data will be quantitatively synthesized by meta-analysis, if appropriate. For BW and BMI outcomes, the effect size of the interventions will be calculated using standardized mean difference or mean difference analyses (95% confidence intervals (95%-CI)) with a fixed- or random-effects meta-analysis (depending on the level of heterogeneity assessed by I^2 statistic or methodology). If important data (standard deviation, post-values, change scores, etc.) is not reported in the included articles, calculations will be performed from the available data (standard error, p-value, 95%-CI, etc.), or we will contact the authors. A narrative synthesis will also be performed to explore the relationship and findings both within and between the included studies for the research questions and sub-analyses.

Discussion

Children and adolescents face significant health challenges today, and above all, two globally-scaled health issues of urgent concern have been identified by health experts who coined overweight/obesity as an „epidemic“ and physical inactivity/insufficient levels of PA as a „crisis“ [20, 47, 56, 57, 116, 139-143, 173]. Since children cannot achieve good health alone, they need support from adults to help them fulfill their potential and thrive [19-21, 24, 112, 144]. Children are key to a nation’s present and future, considering their future roles in raising families and becoming decision- and policy-makers in different settings. Patterns of behavior and lifestyle are established during childhood and adolescence, affecting health for good or bad based on personal choices immediately and in the future [20, 102, 114, 145, 146]. Currently, poor health behavior and the direction of the development of poor health behavior are public health concerns [1, 24, 47, 112, 144, 147].

According to the Global Burden of Disease Study, dietary risks account for 22% of all deaths among adults (≥ 25 years) in Western countries, with more than half of all diet-related deaths linked to low intakes of fruits and whole grains and the high intake of sodium [83, 115]. The five highest-ranked risk factors of premature death worldwide include: (1) hypertension (13%), (2) tobacco use (9%), (3) high blood glucose (6%), (4) physical inactivity (6%), and (5) overweight/obesity (5%) [54-58, 115, 148, 149].

Physical inactivity raises a serious concern because it also supports excess energy intake from unhealthy food products and items [42, 47, 50, 55, 150], but physical inactivity is not the only source of the NCD problem, as NCDs have been shown to develop in highly physically active people [151].

In addition, BMI is related to health, but a person having a BMI within the normal range can still develop other NCDs (e.g., heart disease, cancer, and/or type II diabetes) [1, 33, 34].

Healthy behavior is learned during childhood, and the window for adopting new healthy behaviors diminishes as people grow older [20, 102, 114, 145, 146, 152]. There are tremendous benefits of living a healthy lifestyle, such as the reduced risk for developing NCDs like cancer, diabetes, and heart disease

but also, increased lifespan with a decreased period of disabled years, especially at the end of life [3, 45, 79, 112, 146, 153, 154]. It is, therefore, crucial to teach children a healthy lifestyle as early as possible and offer healthy options in parallel [2, 3, 6, 7, 9, 21, 25, 42, 153-156]. Therefore, early intervention to prevent severe health conditions known to track over time from early childhood into adulthood is key [115].

Although PA is known as an effective tool for improving and shaping good health, PA interventions in schools have been shown to be insufficient to reverse overweight/obesity in the majority of pupils with the disease [44, 101, 103]. More appropriately, without the proper diet, the health benefits of PA are less pronounced [23, 115, 151]. Diet and PA are considered crucial in the fight against overweight/obesity [88], even in children [157], with plant-based diets considered particularly effective to fight overweight/obesity [67, 88, 99, 100, 115, 157-161]. Moreover, information regarding current nutritional trends in children and adolescents (10-19) is limited [23, 115, 162, 163]. To yield maximal health benefits alongside a proper health-promoting diet, PA raises the bar for health even further [1, 3, 6, 7, 9, 10, 23, 110-115, 117, 166, 168].

Diet is very important for health, but permanently linking it with PA, sports, and exercise as another lifestyle factor that is a well-known health tool as a minimum recommendation to achieve sustainable, lifelong health and wellbeing generally creates a permanent linkage from childhood to adulthood with greater cumulative lasting effects [6, 7, 23, 91, 107, 110-112, 115, 146, 169]. This review aims to determine the best practice of PA, dietary, or combined PA and dietary intervention in primary and secondary school pupils for improving BMI and/or BW.

Limitations (3.1)

As school settings are different from clinical settings, it is unknown whether non-randomized study designs will be included in order to address all of our research questions.

Strengths (3.2)

This protocol follows the Preferred Reporting Items for Systematic Review and Meta-Analysis Protocol (PRISMA-P) guidelines [164, 165].

Amendments (3.3)

In the event of protocol amendments, the date of each amendment will be accompanied by a description of the change and the rationale.

Declarations (3.4)

Ethics approval and consent to participate

Not applicable

Consent for publication

Not applicable

Availability of data and material

Not applicable

Competing interests

The authors declare that they have no competing interests.

Funding

This work was supported by the doctoral scholarship University of Innsbruck Vice Rectorate for Research for promoting young scientists: No. 2020/2/PSY/SPORT-21.

Authors' contributions

KW created the inception of the review and DT is the guarantor of the review. KW developed the selection criterion and DT developed the search strategy, risk of bias assessment strategy, data extraction criteria, and performed the systematic review methods with a second reviewer at each step. GR, KW, WK, CD, and TR helped in drafting the manuscript, and GR, CD, DT and KW critically reviewed it. All authors read, provided feedback, and approved the final manuscript.

Acknowledgments

There are no professional relationships with companies or manufacturers who will benefit from the results of the present study. This research did not receive any specific grant or funding from funding agencies in the public, commercial, or not-for-profit sectors.

References

1. World Health Organization. *Global status report on noncommunicable diseases 2010*. Available at: https://www.who.int/nmh/publications/ncd_report_full_en.pdf. [accessed 20.01.2020].
2. Campbell TC & Campbell TM II. *The China study*. Ashland, OR: Blackstone Audio; 2016.
3. Imura H. Life course health care and preemptive approach to non-communicable diseases. *Proceedings of the Japan Academy, Series B Physical and Biologica Sciences* 2013;89(10): 462-473.
4. Jamner MS, Spruijt-Metz D, Bassin S, Cooper DM. A Controlled Evaluation of a School-based Intervention to Promote Physical Activity Among Sedentary Adolescent Females: Project FAB. *Journal of Adolescent Health* 2004;34:279-289.
5. Lynch H, Johnston C, Wharton C. Plant-Based Diets: Considerations for Environmental Impact, Protein Quality, and Exercise Performance. *Nutrients* 2018;10(12): pii: E1841.
6. Lynch J & Smith GD. A life course approach to chronic disease epidemiology. *Annu Rev Public Health* 2005;26:1-35.
7. Mikkelsen B, Williams J, Rakovac I, Wickramasinghe K, Hennis A, Shin HR, Farmer M, Weber M, Berdzuli N, Borges C, Huber M, Breda J. Life course approach to prevention and control of non-communicable diseases. *The BMJ* 2019;364:I257.

8. Parrish A, Okley AD, Stanley RM, Ridgers ND. The Effect of School Recess Interventions on Physical Activity A Systematic Review. *Sports Med* 2013;43:287-299.
9. World Health Organization. *Preventing noncommunicable diseases*. Available at: https://www.who.int/activities/preventing-noncommunicable-diseases. [accessed 19.01.2020].
10. Yuksel H, Sahin FN, Maksimovic N, Drid P, Bianco A. School-Based Intervention Programs for Preventing Obesity and Promoting Physical Activity and Fitness: A Systematic Review. *Int. J. Environ. Res. Public Health* 2020;17(1):347.
11. The World Health Organization. *The top 10 causes of death.* Available at: https://www.who.int/news-room/fact-sheets/detail/the-top-10-causes-of-death. [12.04.2020].
12. The World Health Organization. *Noncommunicable diseases.* Available at: https://www.who.int/news-room/fact-sheets/detail/noncommunicable-diseases. [accessed 29.06.2020].
13. Prynn JE, Kuper H. Perspective on disability and non-communicable diseases in low- and middle-income countries, with a focus on stroke and dementia. *Int J Environ Res Public Health* 2019;16(18):3488.
14. Lichtenstein P, Holm NV, Verkasalo PK, lliado A, Kaprio J, Koskenvuo M, Pukkala E, Skytthe A, Hemminki K. Environmental and heritable factors in the causation of cancer--analyses of cohorts of twins from Sweden, Denmark, and Finland. *N Engl J Med* 2000;343(2):78-85.
15. Sharma M & Majumdar PK. Occupational lifestyle diseases: An emerging issue. *Indian J Occup Environ Med* 2009;13(3):109-112.
16. Willett WC. Balancing life-style and genomics research for disease prevention. *Science (New York)* 2002;296(5568):695-8.
17. Janssen I, Leblanc AG. Systematic review of the health benefits of physical activity and fitness in school-aged children and youth. *Int J Behav Nutr Phys Act* 2010;7:40.
18. Jiménez-Pavón D, Konstabel K, Bergman P, Ahrens W, Pohlabeln H, Hadjigeorgiou C, Siani A, Iacoviello L, Molnar D, De Henauw S, Pitsiladis Y, Moreno L. Physical activity and clustered cardiovascular disease risk factors in young children: a cross-sectional study (the IDEFICS study). *BMC Med* 2013;11:172.
19. Langille JD & Rodgers WM. Exploring the Influence of a Social Ecological Model on School-Based Physical Activity. *Health Educ Behav* 2010;37(6):879-94.
20. Bentham J, di Cesare M, Bilano V, et al. World-wide trends in body-mass index, underweight, overweight, and obesity from 1975 to 2016: a pooled analysis of 2416 population-based measurement studies in 128.9 million children, adolescents, and adults. *Lancet* 2017;390(10113):2627-2642.
21. Currie C, Zanotti C, Morgan A, Currie D, de Looze M, Roberts C, Samdal O, Smith ORF, Barnekow V. eds. Social determinants of health and well-being among young people: Health Behaviour in School-aged Children (HBSC) study: international report from the 2009/2010 survey. (Health Policy for Children and Adolescents, No. 6). 2012. Available at: https://www.euro.who.int/en/countries/armenia/publications/social-determinants-of-health-and-well-being-among-young-people.-health-behaviour-in-school-aged-children-hbsc-study. [accessed 22.01.2020].

22. Schroeder SA. We can do better – Improving the health of the American People. *N Engl J Med* 2007;357:1221-1228.
23. Wirnitzer, K. *Therapeutic, Probiotic, and Unconventional Foods. Chapter 21: Vegan Nutrition: Latest Boom in Health and Exercise.* Elsevier London, UK. (2018).
24. Anselma M, Chinapaw MJM, Altenburg TM. Determinants of child health behaviors in a disadvantaged area from a community perspective: a participatory needs assessment. *Int J Environ Res Public Health* 2018;15(4):644.
25. Grant C. *The Impact of School Health Programmes*. K4D Helpdesk Report. Brighton, UK: Institute of Development Studies. (2017).
26. Brown T & Summerbell C. Systematic review of school-based interventions that focus on changing dietary intake and physical activity levels to prevent childhood obesity: an update to the obesity guidance produced by the National Institute for Health and Clinical Excellence. *Obes Rev* 2009;10:110-141.
27. Wang Y, Cai L, Wu Y, Wilson RF, Weston C, Fawole O, Bleich SN, Cheskin LJ, Showell NN, Lau BD, Chiu DT, Zhang A, Segal J. What childhood obesity prevention programmes work? A systematic review and meta-analysis. *Obes Rev* 2015;16(7):547-565.
28. De Bourdeaudhuij I, Van Cauwenberghe E, Spittaels H, Oppert J-M, Rostami C, Brug J, Van Lenthe F, Lobstein T, Maes L. School-based interventions promoting both physical activity and healthy eating in Europe: a systematic review within the HOPE project. *Obes Rev* 2011;12:205-216.
29. Evans CEL, Albar SA, Vargas-Garcia EJ, Xu F. School-based interventions to reduce obesity risk in children in high- and middle- income countries. *Adv Food Nutr Res* 2015;76:29-77.
30. Flynn MA, McNeil DA, Maloff B, Mutasingwa D, Wu M, Ford C, Tough SC. Reducing obesity and related chronic disease risk in children and youth: a synthesis of evidence with 'best practice' recommendations. *Obes Rev* 2006;7S:17-66.
31. Garcia-Hermoso A, Alonso-Martinez AM, Ramirez-Velez R, Perez-Sousa MA, Ramirez-Campillo R, Izquierdo M. Association of physical education with improvement of health-related physical fitness outcomes and fundamental motor skills among youths. A systematic review and meta-analysis. *JAMA Pediatrics* 2020;174(6):e200223.
32. Mantjes JA, Jones AP, Corder K, et al. School related factors and 1yr change in physical activity amongst 9-11 year old English schoolchildren. *Int J Behav Nutr Phys Act* 2012;9:153.
33. Khan SS, Ning H, Wilkins JT, Allen N, Carnethon M, Berry JD, Sweis RN, Lloyd-Jones DM. Association of body mass index with lifetime risk of cardiovascular disease and compression of morbidity. *JAMA Cardiol* 2018;3(4):280-287.
34. Steele CB, Thomas CC, Henley SJ, Massetti GM, Galuska DA, Agurs-Collins T, Puckett M, Richardson LC. Vital Signs: trends in incidence of cancers associated with overweight and obesity — United States, 2005–2014. *MMWR Morb Mortal Wkly Rep* 2017;66(39):1052–1058.
35. Freedman DS, Dietz WH, Srinivasan SR, Berenson GS. The relation of overweight to cardiovascular risk factors among children and adolescents: the Bogalusa Heart Study. *Pediatrics* 1999;103(6):1175-1182.

36. The World Health Organization. *Body mass index – BMI.* Available at: https://www.euro.who.int/en/health-topics/disease-prevention/nutrition/a-healthy-lifestyle/body-mass-index-bmi. [accessed 02.07.2020]
37. Ruedl G, Ewald P, Niedermeier M, Kirschner W, Kopp M, Drenowatz C, Greier K. Long-term effect of migration background on the development of physical fitness among primary school children. *Scand J Med Sci Sports* 2018;1-8.
38. Centers for Disease Control and Prevention. *Body Mass Index (BMI).* Available at: https://www.cdc.gov/healthyweight/assessing/bmi/index.html. [accessed 10.04.2020]
39. The World Health Organization. *BMI-for-age (5-19 years).* Available at: https://www.who.int/growthref/who2007_bmi_for_age/en/. [accessed 02.07.2020]
40. Freedman DS & Sherry B. The validity of BMI as an indicator of body fatness and risk among children. *Pediatrics* 2009;124(S1):23–34.
41. The Global BMI Mortality Collaboration. Body-mass index and all-cause mortality: individual participant-data meta-analysis of 239 prospective studies in four continents. *Lancet* 2016;388(10046):P776-786.
42. Yoo S. Dynamic energy balance and obesity prevention. *J Obes Metab Syndr* 2018;27(4):203-212.
43. Fairclough S & Strattona G. Effects of a physical education intervention to improve student activity levels. *Physical Education and Sports Pedagogy* 2006;11(1):29-44.
44. Metcalf B, Henley W, Wilkin T. Effectiveness of intervention on physical activity of children: systematic review and meta-analysis of controlled trials with objectively measured outcomes (EarlyBird 54). *The BMJ* 2012;345:e5888.
45. Fredriksen PM, Hjelle OP, Mamen A, Meza TJ, Westerberg A.C. The health oriented pedagogical project (HOPP) a controlled longitudinal school-based physical activity intervention program. *BMC Public Health* 2017;17:370.
46. Timmons BW, Proudfoot NA, MacDonald MJ, Bray S, Steven, Cairney J. The health outcomes and physical activity in preschoolers (HOPP) study: rationale and design. *BMC Public Health* 2012;12(1):284.
47. Blair SN. Physical inactivity: the biggest public health problem of the 21st century. *Br J Sports Med* 2009;43:1- 2.
48. Hills AP, Okely AD, Baur LA. Addressing childhood obesity through increased physical activity. *Nat Rev Endocrinol* 2010;6(10):543-549.
49. Booth FW, Roberts CK, Laye MJ. Lack of exercise as a major cause of chronic disease. *Compr Physiol* 2012;2(2):1143-1211.
50. Hespanhol LC jr., Pillary JD, van Mechelen W, Verhagen E. Meta-analyses of the effects of habitual running on indices of health in physically inactive adults. *Sports Med* 2015;45(10):1455-1468.
51. Wen CP, Wai JPM, Tsai MK, Yang YC, Cheng TYD, Lee MC, Chan HT, Tsao CK, Tsai SP, Wu X. Minimum amount of physical activity for reduced mortality and extended life expectancy: a prospective cohort study. *The Lancet* 2011;378:1244-53.

52. Ring-Dimitriou S, von Duvillard SP, Paulweber B, Stadlmann M, LeMura LM, Peak K, Mueller E. Nine months aerobic fitness induced changes on blood lipids and lipoproteins in untrained subjects versus controls. *Eur J Appl Physiol* 2007;99:291-299.
53. Lima RA, Andersen LB, Soares FC, Kriemler S. The causal pathways effects of a physical activity intervention on adiposity in children: The KISS study cluster randomized clinical trial. *Scand J Med & Sci Sports* 2020;00:1-7.
54. The World Health Organization. G*lobal recommendations on physical activity for health 9-10.* Available at: https://www.who.int/dietphysicalactivity/global-PA-recs-2010.pdf. [accessed 07.08.2020].
55. Euractiv-Special-Report. *Physical inactivity costs estimated at € 80 bn per year.* Available at: https://www.euractiv.com/section/sports/news/physical-inactivity-costs-estimated-at-80bn-per-year/. [accessed 12.09.2020].
56. The World Health Organization. G*lobal recommendations on physical activity for health 7-8.* Available at: https://www.who.int/dietphysicalactivity/global-PA-recs-2010.pdf. [accessed 07.08.2020].
57. The World Health Organization. G*lobal recommendations on physical activity for health 15-34.* Available at: https://www.who.int/dietphysicalactivity/global-PA-recs-2010.pdf. [accessed 07.08.2020].
58. The World Health Organization. *Physical activity strategy for the WHO European region.* Regional committee for Europe 65th session. Available at: https://www.euro.who.int/__data/assets/pdf_file/0010/282961/65wd09e_PhysicalActivityStrategy_150474.pdf. [accessed 24.03.2020]
59. Department of Health and Human Services, Washington, DC: USA. *2018 Physical Activity Guidelines Advisory Committee Scientific Report.* Available at: https://health.gov/sites/default/files/2019-09/PAG_Advisory_Committee_Report.pdf. [accessed 22.05.2020].
60. Amit M. Vegetarian diets in children and adolescents. *Paediatr Child Health* 2010;15(5):303-314.
61. Barnard ND, Goldman DM, Loomis JF, Kahleova H, Levin SM, Neabore S, Batts TC. Plant-Based Diets for Cardiovascular Safety and Performance in Endurance Sports. *Nutrients* 2019;11(1):pii:E130.
62. Newby PK. Plant foods and plant-based diets: protective against childhood obesity? The American Journal of Clinical Nutrition 2009;89(5):1572S-1587S.
63. Orlich MJ, Jaceldo-Siegl K, Sabaté J, Fan J, Singh PM, Fraser GE. Patterns of food consumption among vegetarians and non-vegetarians. *Br J Nutr* 2014;112(10):1644-1654.
64. Sabaté J & Wien M. Vegetarian diets and childhood obesity prevention. *The American Journal of Clinical Nutrition* 2010;91(5):1525S-1529S.
65. Weder S, Hoffmann M, Becker K, Alexy U, Keller M. Energy, Macronutrient Intake, and Anthropometrics of Vegetarian, Vegan, and Omnivorous Children (1-3 Years) in Germany (VeCHi Diet study). *Nutrients* 2019;11:832.
66. Wirnitzer K, Boldt P, Lechleitner C, Wirnitzer G, Leitzmann C, Rosemann T, Knechtle B. Health Status of Female and Male Vegetarian and Vegan Endurance Runners Compared to Omnivores-Results from the NURMI Study (Step 2). *Nutrients* 2018;11(1):pii:E29.

67. Spencer EA, Applyby PN, Davey GK, Key TJ. Diet and body mass index in 38000 Epic-Oxford meat-eaters, fish-eaters, vegetarians and vegans. *Int J Obes Relat Metab Disord* 2003;27(6):728-34.
68. Gomez-Donoso C, Martinez-Gonzales MA, Martinez JA, Gea A, Sanz-Serrano J, Perez-Cueto FJA, Bes-Rastrollo M. A provegetarian food pattern emphasizing preference for healthy plant-derived foods reduces the risk of overweight/obesity in the SUN cohort. *Nutrients* 2019;11(7)pii: E1553.
69. Appleby PN & Key TJ. The long-term health of vegetarians and vegans. *Proc Nutr Soc* 2016;75(3)287-93.
70. Key TJ, Appleby PN, Crowe FL, Bradbury KE, Schmidt JA, Travis RC. Cancer in British vegetarians: updated analyses of 4998 incident cancers in a cohort of 32,491 meat eaters, 8612 fish eaters, 18,298 vegetarians, and 2246 vegans. *Am J Clin Nutr* 2014;1:378S-85S.
71. Orlich MJ, Singh PM, Sabaté J, Jaceldo-Siegl K, Fan J, Knutsen, Beeson WL, Fraser GE. Vegetarian dietary patterns and mortality in adventist health study 2. *JAMA Intern Med* 2014;173(13):1230-1238.
72. Tonstad S, Stewart K, Oda K, Batech M, Herring RP, Fraser GE. Vegetarian diets and the risk of diabetes in the adventist health study-2. *Nutr Metab Cardiovasc Dis* 2013;23(4):292-9.
73. Le LT & Sabaté J. Beyond meatless, the health effects of vegan diets: findings from the adventist cohorts. *Nutrients* 2014;6:2131-2147.
74. Rizzo NS, Jaceldo-Siegl K, Sabaté J, Fraser GE. Vegetarian dietary patterns are associated with a lower risk of metabolic syndrome. The adventist health study 2. *Diabetes care* 2011;34:1225-1227.
75. Beeson WL, Mills PK, Phillips RL, Andress M, Fraser GE. Chronic disease among seventh-day adventists, a low-risk group. *Cancer* 1989;64:570-581.
76. Chiu TH, Huang HY, Chiu YF, Pan WH, Kao HY, Chiu JP, Lin MN, Lin CL. Taiwanese vegetarians and omnivores: dietary composition, prevalence of diabetes and IFG; PLoS One. 2014;9(2):e88547.
77. Li D. Effect of the vegetarian diet on non-communicable diseases. *J Sci Food Agric* 2014;94(2):169-173.
78. Cullum-Dugan D & Pawlak R. Position of the academy of nutrition and dietetics: vegetarian diets. *J Acad Nutr Diet* 2015;115(5):801-10.
79. Tuso P. Physician update: total health. *PermJ* 2014;18(2):58-63.
80. Tuso P, Ismail M, Ha B, Bartolotto C. Nutritional update for physicians: plant based diets. *PermJ* 2015;17(2):61-66.
81. Tuso P, Stoll SR, Li WW. A plant-based diet, atherogenesis, and coronary artery disease prevention. *PermJ* 2015;19(1):62-67.
82. Melina V, Craig W, Levin S. Position of the academy of nutrition and dietetics: vegetarian diets. *J Acad Nutr Diet.* 2016;116(12):1970-1980.
83. GBD-Diet-Collaborators. Health effects of dietary risks in 195 countries, 1900-2017: a systematic analysis for the global burden of disease study 2017. *Lancet* 2019;393:1958-1972.
84. Esselstyn CB Jr. Resolving the Coronary Artery Disease Epidemic Through Plant-Based Nutrition. *Prev Cardiol 2001;*4(4):171-177.
85. Esselstyn CB Jr. *Prevent and Reverse Heart Disease. The Revolutionary, Scientifically Proven, Nutrition-Based Cure.* London: Penguin Books; 2008.

86. Esselstyn CB Jr., Gendy G, Doyle J, Golubic M, Roizen MF. A way to reverse CAD? *J Fam Pract* 2014;63(7): 356-364b.
87. Kadoch M. The power of nutrition as medicine. *Prev Med* 2012;55(1):80.
88. Katz DL. Unfattening our children: forks over feet. *Int J Obes* 2011;35:33-37.
89. Ornish D. *Dr. Dean Ornish΄s Program for Reversing Heart Disease: The Only System Scientifically Proven to Reverse Heart Disease Without Drugs or Surgery.* New York: Ivy Books; 1996
90. Ornish D, Schwerwitz L, Billings J, Gould L, Merritt T, Sparler S, Armstrong W, Ports T, Kirkeeide R, Hogeboom C, Brand R. Intensive Lifestyle changes for reversal of coronary heart disease. *JAMA* 1998;280(23):2001-2007.
91. Ardisson Korat AV, Willett WC, Hu FB. Diet, lifestyle, and genetic risk factors for type 2 diabetes: a review from the Nurses' Health Study, Nurses' Health Study 2, and Health Professionals' Follow-up Study. *Curr Nutr Rep* 2014;3(4):345-54.
92. Willcox BJ, Willcox DC, Todoriki H, Fujiyoshi A, Yano K, He Q, Curb D, Suzuki M. Caloric restriction, the traditional Okinawan diet, and healthy aging. The diet of the world’s longest lived peopleand its potential impact on morbidity and life span. *Ann N. Y. Acad Sci* 2007;1114:435-455.
93. Willcox DC, Willcox BJ, Todoriki H, Suzuki M. The Okinawan diet: health implications of a low-calorie, nutrient-dense, antioxidant-rich, dietary pattern low in glycemic load. *J Am Coll Nutr* 2009;(28S):500-516.
94. Willcox DC, Scapagnini G, Willcox BJ. Healthy aging diets other than the Mediterranean: a focus on the Okinawan diet. *Mech Ageing Dev* 2014;136-137:148-162.
95. Ornish D, Brown SE, Scherwitz LW, Billings JH, Armstrong WT, Ports TA, McLanahan SM, Kirkeeide RL, Brand RJ, Gould KL. Can lifestyle changes reverse coronary heart disease? *The Lancet* 1990;336:129-133.
96. Ornish D, Weidner G, Fair WR, Marlin R, Pettengil EB, Rasin CJ, Dunn-Emke S, Crutchfield L, Jacobs FN, Barnard RJ, Aronson WJ, McCormac O, McKnight DJ, Fein JD, Dnistrain AM, Weinstein J, Ngo TH, Mendell NR, Carroll PR. Intensive lifestyle changes may affect the progression of prostate cancer. *The Journal of Urology* 2005;174:1065-1070.
97. Esselstyn Jr CB, Gendy G, Doyle J, Golubic M, Roizen MF. A way to reverse CAD? *The Journal of Family Practice* 2014;63(7):356-364.
98. McMacken M & Shah S. A plant-based diet for the prevention and treatment of type 2 diabetes. *J Geriatr Cardiol* 2017;14(5):342-354.
99. Key T & Davey G. Prevalence of obesity is low in people who do not eat meat. *BMJ* 1996;313:816-7.
100. Berkow SE & Barnard N. Vegetarian diets and weight status. *Nutr Rev* 2006;64:175-88.
101. Webber LS, Catellier DJ, Lytle LA, Murray DM, Pratt CA, Young DR, Elder JP, Lohman TG, Stevens J, Jobe JB, Pate RR. Promoting Physical Activity in Middle School Girls Trial of Activity for Adolescent Girls. *American Journal of Preventative Medicine* 2008;34(3):173-184.
102. Ruedl G, Greier N, Niedermeier M, Posch M, Prünster V, Faulhaber M, Burtscher M. Factors Associated with Physical Fitness among Overweight and Non-Overweight Austrian Secondary School Students. *International Journal of Environmental Research and Public Health* 2019;16:4117.

103. Ruedl G, Franz D, Frühauf A, Kopp M, Niedermeier M, Drenowatz C, Greier K. Development of physical fitness in Austrian primary school children. A longitudinal study among overweight and non-overweight children over 2.5 years. *Wien Klin Wochenschr* 2018;130:321-327.
104. Ruedl G, Greier K, Kirschner W, Kopp M. Factors associated with motor performance among overweight and nonoverweight Tyrolean primary school children. *Wien Klin Wochenschr* 2015;128(1).
105. Drenowatz C, Greier K, Ruedl G, Kopp M. Association between Club Sports Participation and Physical Fitness across 6- to 14-Year-Old Austrian Youth. *International Journal of Environmental Research and Public Health* 2019;16:3392.
106. Greier K, Ruedl G, Weber C, Thöni G, Riechelmann H. Eating behavior and motor performance of 10 to 14 year old adolescents (Ernährungsverhalten und motorische Leistungsfähigkeit von 10- bis 14-jährigen Jugendlichen). Ernährung und Medizin 2016;31(4). [in German].
107. Adom T, De Villiers A, Puoane T, Kengne AP. School-based interventions targeting nutrition and physical activity, and body weight status of African children: a systematic review. *Nutrients* 2019;12(1)pii:E95.
108. Singhal J, Herd C, Adab P, Pallan M. Effectiveness of school-based interventions to prevent obesity among children aged 4 to 12 years in middle-income countries: A systematic review and meta-analysis. *Obesity Reviews* 2020;1-39.
109. Grydeland M, Bergh IH, Bjelland M, Lien N, Andersen LF, Ommundsen Y, Klepp KI, Anderssen SA. Intervention effects on physical activity: the HEIA study - a cluster randomized controlled trial. *International Journal of Behavioral Nutrition and Physical Activity* 2013;10:17.
110. Voráčová J, Badura P, Hamrik Z, Holubčíková J, Sigmund E. Unhealthy eating habits and participation in organized leisure-time activities in Czech adolescents. *European Journal of Pediatric* 2018;177:1505-1513.
111. Thivel D, Tremblay MS, Katzmarzyk PT, Fogelholm M, Hu G, Maher C, Maia J, Olds T, Sarmiento OL, Standage M, Tudor-Locke C, Chaput JP. Associations between meeting combinations of 24-hours movement recommendations and dietary patterns of children: A 12-country study. *Preventive Medicine* 2019;118:159-165.
112. Santaliestra-Pasías AM, Llamas Dios JE, Sprengeler O, Hebestreit A, De Henauw S, Eiben G, Felsö R, Lauria F, Tornaritis M, Veidebaum T, Pala V, Moreno LA. Food and beverage intakes according to physical activity levels in European children: the IDEFICS (Identification and prevention of Dietary and lifestyle induced health EFfects In Children and infantS) study. *Public Health Nutrition* 2018;21(9):1717-1725.
113. Galan-Lopez P, Domínguez R, Pihu M, Gísladóttir T, Sánchez-Oliver AJ, Reis F. Evaluation of Physical Fitness, Body Composition, and Adherence to Mediterranean Diet in Adolescents from Estonia: The AdolesHealth Study. *International Journal of Environmental Research and Public Health* 2019;16:4479.
114. Drenowatz C & Grier K. Association of Sports Participation and Diet with Motor Competence in Austrian Middle School Students. *Nutrients* 2018;10:1837.
115. Wirnitzer K. Vegan diet in sports and exercise – health benefits and advantages to athletes and physically active people: a narrative review. *Int J Sports Exerc Med* 2020;6:165.

116. Yáñez-Ortega JL, Arrieta-Cerdán E, Lozano-Alonso JE, Gil Costa M, Gutiérrez-Araus AM, Cordero-Guevara JA, Vega Alonso T. Grupo de Trabajo para la Investigación de Sobrepeso, Obesidad y Desarrollo Infantil en Castilla y León. Prevalence of overweight and obesity in child population. A study of a cohort in Castile and Leon, Spain. *Endocrinol Diabetes Nutr* 2019;66(3):173-180.
117. Williams PT. Interactive effects of exercise, alcohol, and vegetarian diet on coronary artery disease risk factors in 9242 runners: the National Runners' Health Study. *Am J Clin Nutr* 1997;66(5):1197-1206.
118. Haerens L, Deforche B, Maes L, Stevens V, Cardon G, De Bourdeaudhuij I. Body mass effects of a physical activity and healthy food intervention in middle schools. *Obesity* 2006;14:847–854.
119. Norris E, van Steen T, Direito A, Stamatakis E. Physical activity lessons in schools and their impact on physical activity, educational, health, and cognition outcomes: a systematic review and meta-analysis. *Br J Sports Med* 2019;54:826-838.
120. Dobbins M, Husson H, DeCorby K, et al. School-Based physical activity programs for promoting physical activity and fitness in children and adolescents aged 6 to 18. *Cochrane Database Syst Rev* 2013;19.
121. Bartholomew JB & Jowers EM. Physically active academic lessons in elementary children. *Prev Med* 2011;52(S1):51–54.
122. Riley N, Lubans DR, Morgan PJ, et al. Outcomes and process evaluation of a programme integrating physical activity into the primary school mathematics curriculum: the easy minds pilot randomised controlled trial. *J Sci Med Sport* 2015;18:656–61.
123. Norris E, Shelton N, Dunsmuir S, et al. Physically active lessons as physical activity and educational interventions: a systematic review of methods and results. *Prev Med* 2015;72:116–25.
124. de Greeff JW, Hartman E, Mullender-Wijnsma MJ, et al. Effect of physically active academic lessons on body mass index and physical fitness in primary school children. *J Sch Health* 2016;86:346–52.
125. Ridgers ND, Timperio A, Cerin E, et al. Compensation of physical activity and sedentary time in primary school children. *Med Sci Sports Exerc* 2014;46:1564–9.
126. Naylor P-J, Nettlefold L, Race D, et al. Implementation of school based physical activity interventions: a systematic review. *Prev Med* 2015;72:95–115.
127. Kobes A, Kretschmer T, Timmerman G, Schreuder P. Interventions aimed at preventing and reducing overweight/obesity among children and adolescents: a meta- synthesis. *Obes Rev* 2018;19:1065- 1079.
128. Harris KC, Kuramoto LK, Schulzer M, Retallack JE. Effect of school- based physical activity interventions on body mass index in children: a meta- analysis. *Can Med Assoc J* 2009;180:719- 726.
129. Guerra P, Nobre M, Silveira J, Taddei J. The effect of school- based physical activity interventions on body mass index: a meta- analysis of randomized trials. *Clinics* 2013;68:1263- 1273.
130. Hung L- S, Tidwell DK, Hall ME, Lee ML, Briley CA, Hunt BP. A meta- analysis of school- based obesity prevention programs demonstrates limited efficacy of decreasing childhood obesity. *Nutr Res* 2015;35:229-240.

131. Klakk H, Chinapaw M, Heidemann M, Andersen LB, Wedderkopp N. Effect of four additional physical education lessons on body composition in children aged 8-13 years: a prospective study during two school years. *BMC Pediatr* 2013;13:170.
132. Singhal J, Herd C, Adab P, Pallan M. Effectiveness of school-based interventions to prevent obesity among children aged 4 to 12 years in middle-income countries: A systematic review and meta-analysis. *Obesity Reviews* 2020;1-39.
133. Reeves BC, Deeks JJ, Higgins JPT, Shea B, Tugwell P, Wells GA. *Cochrane Handbook for Systematic Reviews of Interventions (version 6.0).* Chapter 24: Including non-randomized studies on intervention effects. Cochrane, 2019. Available at: www.training.cochrane.org/handbook.
134. Caspersen CJ, Powell KE, Christenson GM. Physical activity, exercise, and physical fitness: definitions and distinctions for health-related research. *Public Health Reports* 1985;100(2):126-131.
135. Clarys P, Deliens T, Huybrechts I, Deriemaeker P, Vanaelst B, De Keyzer W, Hebbelinck M, Mullie P. Comparison of nutritional quality of the vegan, vegetarian, semi-vegetarian, pesco-vegetarian, and omnivorous diet. *Nutrients* 2014;6(3):1318-1332.
136. The Cochrane Collaboration. *Cochrane Handbook for Systematic Reviews of Interventions (version 6.0).* Available at: www.training.cochrane.org/handbook.
137. Sterne JAC, Hernán MA, McAleenan A, Reeves BC, Higgins JPT. *Cochrane Handbook for Systematic Reviews of Interventions (version 6.0).* Chapter 25: Assessing risk of bias in a non-randomized study. Available at: www.training.cochrane.org/handbook.
138. Higgins JPT, Altman DG, Gøtzsche PC, et al. The Cochrane collaboration's tool for assessing risk of bias in randomised trials. *BMJ* 2011;343:d5928.
139. Lobstein T, Baur L, Uauy R. Obesity in children and young people: a crisis in pubic health. *Obesity Reviews* 2004;5(S1):4-85.
140. Griffiths LJ, Cortina-Borja M, Sera F, et al. How active are our children? findings from the millennium cohort study. *BMJ Open* 2013;3:e002893.
141. Hallal PC, Andersen LB, Bull FC, et al. Global physical activity levels: surveillance progress, pitfalls, and prospects. *The Lancet* 2012;380:247–57.
142. Bailey DP, Fairclough SJ, Savory LA, et al. Accelerometry-assessed sedentary behaviour and physical activity levels during the segmented school day in 10-14-year-old children: the happy study. *Eur J Pediatr* 2012;171:1805–13.
143. di Cesare M, Sorić M, Bovet P, Miranda JJ, Bhutta Z, Stevens G, Laxmaiah A, Kengne AP, Bentham J. The epidemiological burden of obesity in childhood: A worldwide epidemic requiring urgent action. *BMC Med* 2019;17:1–20.
144. Institute of Medicine (US) Committee on Assuring the Health of the Public in the 21st Century. *The Future of the Public's Health in the 21st Century.* Washington (DC): National Academies Press (US); 2002. Chapter 2: Understanding Population health and Its Determinants. Available at: https://www.ncbi.nlm.nih.gov/books/NBK221239/. doi: 10.17226/10548 [accessed 02.12.2020].

145. Greier K, Drenowatz C, Ruedl G, Riechelmann H. Association between daily TV time and physical fitness in 6- to 14-year-old Austrian youth. *Translational Pediatrics* 2019;8(5):371-377.

146. Moreno LA, Gottrand F, Huybrechts I, Ruiz JR, González-Gross M, DeHenauw S. Nutrition and Lifestyle in European Adolescents: The HELENA (Healthy Lifestyle in Europe by Nutrition in Adolescence) Study. *Advances in Nutrition* 2014;5(5S):615-623.

147. Marques A, Peralta M, Naia A, Loureiro N, de Matos MG. Prevalence of Adult overweight and obesity in 20 European countries, 2014. *The European Journal of Public Health* 2017;1-5.

148. The World Health Organization. *Global Health Risks: Mortality and Burden of Disease Attributable to Selected Major Risks.* 2009. Available at: https://www.who.int/healthinfo/global_burden_disease/GlobalHealthRisks_report_full.pdf. [accessed 09.10.2020]

149. Beaglehole R, Bonita R, Alleyne G, Horton R, Li L, et al. UN High-Level meeting on non-communicable diseases: addressing four questions. *Lancet* 2011;378:449-455.

150. Lee IM, Shiroma EJ, Lobelo F, Puska P, Blair SN, Katzmarzyk PT. Effect of physical inactivity on major non-communicable diseases worldwide: an analysis of burden of disease and life expectancy. *Lancet* 2012;380:219-229.

151. Enos WF Jr., Beyer JC, Holmes RH. Pathogenesis of coronary disease in American soldiers killed in Korea. *JAMA* 1995;158(11):912-914.

152. Michie S, Richardson M, Johnston M, et al. The behavior change technique taxonomy (V1) of 93 hierarchically clustered techniques: building an international consensus for the reporting of behavior change interventions. *Ann Behav Med* 2013;46:81–95.

153. US Burden of Disease Collaborators. The state of US health, 1990–2010: burden of diseases, injuries, and risk factors. *JAMA* 2013;310:591–608.

154. Willcox BJ, Suzuki M, Donlon TA, He Q, Grove JS, Masaki K, Willcox DC. Optimizing human health span: insights from Okinawa and Hawaii. *Annu Rev Gerontol Geriatr* 2013;33(1):133-170.

155. Wirnitzer K. From Science 2 School: Sustainably healthy - active & veggie. Research sketch. transfer research <> school, booklet (Nachhaltig gesund – bewegt & veggie. Forschungsskizze. transfer Forschung <> Schule, Heft) 2019;5:S241-243. [in German].

156. Physicians Committee for Responsible Medicine. *Nutrition for Kids. A Dietary Approach of Lifelong Health (2018-e).* Available at: http://www.pcrm.org/sites/default/files/pdfs/health/Nutrition_for_Kids.pdf. [accessed [06.07.2020].

157. Vorwerg Y, Petroff D, Kiess W, Blueher S. Physical activity in 3 – 6 year old children measured by SenseWear Pro*: direct accelerometry in the course of the week and relation to weight status, media consumption, and socioeconomic factors. *PLoS one* 2013;8e60619.

158. Kahleova H, Levin S, Barnard N. Cardio-metabolic benefits of plant-based diets. *Nutrients* 2017;9(8):848.

159. Physicians Committee for Responsible Medicine. *Plant-Based Diets. The power of a plant-based diet for good health.* 2021. Available at: https://www.pcrm.org/good-nutrition/plant-based-diets. [accessed 12.01.2021].

160. Barnard N, Levin SM, Yokoyama Y. A systematic review and meta-analysis of changes in body weight in clinical trials of vegetarian diets. *J Acad Nutr Diet* 2014;2212-2672.

161. Barnard N, Kahleova H, Levin SM. The use of plant-based diets for obesity treatment. *International Journal of Diease Reversal and Prevention* 2019;2(1):12pp.

162. Ipsos Retail Performance. (2020). *Exploring the explosion of veganism in the United States.* Available at: https://www.ipsos-retailperformance.com/en/vegan-trends/. [accessed 03.10.2020]

163. Chiorando M. *30% of British shoppers aged 18-24 are vegan or considering it, says poll. An increasing number of young people are ditching animal products.* 2018. Available at: https://www.plantbasednews.org/culture/30-british-shoppers-18-24-vegan-considering. [accessed 03.10.2020].

164. Moher D, Shamseer L, Clarke M, Ghersi D, Liberati A, Petticrew M, Shekelle P, Stewart LA. Preferred Reporting Items for Systematic Review and Meta-Analysis Protocols (PRISMA-P) 2015 statement. *Syst Rev* 2015;4(1):1.

165. Shamseer L, Moher D, Clarke M, Ghersi D, Liberati A, Petticrew M, Shekelle P, Stewart L, PRISMA-P Group. Preferred reporting items for systematic review and meta-analysis protocols (PRISMA-P) 2015: elaboration and explanation. *BMJ* 2015;349(1):g7647.

166. Godoy-Cumillaf A, Fuentes-Merino P, Diaz-Gonzalez A, Jimenez-Diaz J, Martinez-Vizcaino V, Alvarez-Bueno C, Cavero-Redondo I. The effects of physical activity and diet interventions on body mass index in Latin American children and adolescents: a systematic review and meta-analysis. *Nutrients* 2020;12:1378.

167. Furtado A. Monitoring the activities of the EU platform on diet, physical activity and health. Annual Report European Commission. 2015;1-144. Available at: https://ec.europa.eu/health/sites/default/files/nutrition_physical_activity/docs/2015_report_en.pdf. [accessed 23.07.2021].

168. European Commission. Nutrition and physical activity. 2021 Available at: https://ec.europa.eu/health/nutrition_physical_activity/overview_en. [accessed 23.07.2021].

169. Independent Accountability Panel. The health of women, children and adolescents is at the heart of transforming our world: empowering accountability. Final reflections report 2021;1-26. Available at: https://iapewec.org/reports/iap-2021-final-report/. [accessed 23.07.2021].

170. Sweet SN & Fortier MS. Improving physical activity and dietary behaviors with single or multiple health behavior interventions? A synthesis of meta-analyses and reviews. *Int J Environ. Res. Public Health* 2010;7:1720-1743.

171. Lewis JE & Schneiderman N. Nutrition, physical activity, weight management, and health. *Revista Colombiana de Psiquiatria* 2006; Supplemento, vol. XXXV.

172. Baranowski T. Why combine diet and physical activity in the same international research? *International Journal of Behavioral Nutritional and Physical Activity* 2004;1:2.

173. The Wold Health Organization. Report of the commission on ending childhood obesity. Implementation plan: executive summary. 2017;1-24. Available at: https://apps.who.int/iris/bitstream/handle/10665/259349/WHO-NMH-PND-ECHO-17.1-eng.pdf. [accessed 23.07.2021].

174. Fjeldsoe B, Neuhaus M, Winkler E, Eakin E. Systematic review of maintenance of behavior change following physical activity and dietary interventions. *Health Psychology* 2011;30(1):99-109.

Click here to access/download
Supporting Information
PRISMA-P-checklist.doc
