# Supplementary material for: School health programs of physical education and/or diet among pupils of primary and secondary school levels I and II linked to body mass index: A systematic review protocol within the project From Science 2 School
Source: PLoS One. 2022 Oct 6;17(10):e0275012. doi: 10.1371/journal.pone.0275012 (PMC9536596; doi:10.1371/journal.pone.0275012)
Supplement: Attachment — Submitted filename: Response to Reviewer 3_PONE-D-21-25090.docx [file pone.0275012.s003.docx]

Dear Reviewer #3,

thank you for your consideration, positive feedback, and the opportunity to revise the manuscript *PONE-D-21-25090* entitled “*School health programs of physical education and/or diet among pupils of primary and secondary school levels I and II in relation to body mass index: a systematic review protocol within the project From Science 2 School*”, which has led to a significant improvement of the manuscript.

In response to your valuable comments, we provided some evidence along with detailed explanations that directly address each comment. We hope our responses below and revisions throughout the manuscript will satisfy the existing concerns of the reviewer and editors. Changes in the manuscript have been highlighted via “Track Changes”.

Kind Regards,

Derrick Tanous and Katharina Wirnitzer, on behalf of the team of authors

**Review Report 3**

-----------------------------------------------------------------------------------------------------------------------------

**Comments mentioned by Reviewer #3 (in blue) and our responses to each comment (in red)**

-----------------------------------------------------------------------------------------------------------------------------

1. Does the manuscript provide a valid rationale for the proposed study, with clearly identified and justified research questions?

   The research question outlined is expected to address a valid academic problem or topic and contribute to the base of knowledge in the field.

Reviewer #3: Yes

Answer: Thank you – we appreciate your kind feedback and consideration.

2. Is the protocol technically sound and planned in a manner that will lead to a meaningful outcome and allow testing the stated hypotheses?

   The manuscript should describe the methods in sufficient detail to prevent undisclosed flexibility in the experimental procedure or analysis pipeline, including sufficient outcome-neutral conditions (e.g. necessary controls, absence of floor or ceiling effects) to test the proposed hypotheses and a statistical power analysis where applicable. As there may be aspects of the methodology and analysis which can only be refined once the work is undertaken, authors should outline potential assumptions and explicitly describe what aspects of the proposed analyses, if any, are exploratory.

Reviewer #3: Yes

Answer: Thank you very much for your positive consideration regarding the meaningful systematic review and meta-analysis outcome to identify the best practice of PA, dietary, or combined PA and dietary intervention in primary and secondary school pupils for improving BMI and/or BW.

3. Is the methodology feasible and described in sufficient detail to allow the work to be replicable?

   Descriptions of methods and materials in the protocol should be reported in sufficient detail for another researcher to reproduce all experiments and analyses. The protocol should describe the appropriate controls, sample size calculations, and replication needed to ensure that the data are robust and reproducible.

Reviewer #3: Yes

Answer: Thank you – we appreciate your thoughtful feedback very much, especially due to the thorough efforts planning this investigation’s methodology following PRISMA standards.

4. Have the authors described where all data underlying the findings will be made available when the study is complete?

   The [PLOS Data policy](https://journals.plos.org/plosone/s/materials-and-software-sharing) requires authors to make all data underlying the findings described in their manuscript fully available without restriction, with rare exception, at the time of publication. The data should be provided as part of the manuscript or its supporting information, or deposited to a public repository. For example, in addition to summary statistics, the data points behind means, medians and variance measures should be available. If there are restrictions on publicly sharing data—e.g. participant privacy or use of data from a third party—those must be specified.

Reviewer #3: Yes

Answer: Thank you – we value your attentive consideration.

5. Is the manuscript presented in an intelligible fashion and written in standard English?

   PLOS ONE does not copyedit accepted manuscripts, so the language in submitted articles must be clear, correct, and unambiguous. Any typographical or grammatical errors should be corrected at revision, so please note any specific errors here.

Reviewer #3: Yes

Answer: Thank you – we appreciate the rating of the expert reviewer.

6. Review Comments to the Author

   Please use the space provided to explain your answers to the questions above and, if applicable, provide comments about issues authors must address before this protocol can be accepted for publication. You may also include additional comments for the author, including concerns about research or publication ethics.

   You may also provide optional suggestions and comments to authors that they might find helpful in planning their study.

Reviewer #3:

The aim of this manuscript is to highlight the different approaches (physical intervention, nutritional intervention, and dual-approach of diet and exercise) and identify effective intervention s for sustainable body weight and healthy body mass index in school children. The aim of the work is set clearly and intelligibly. The introduction is clear and theory based. The research methodology is set correctly for this type of work (PRISMA guidlines). Although the conclusions do not bring new signoificant findings, the authors make an interesting contribution to the literature in this research area.

Answer: Thank you – we highly appreciate your kind feedback, thorough understanding, and consideration of the manuscript. As this article is a protocol designed to peak the accuracy, transparency, frequency, and completeness of systematic review and meta-analysis methodology, there are no results to the current manuscript to draw conclusions with new significance, however, the results and conclusions of the follow-up systematic review parts 1 (secondary schools) and 2 (primary schools) will contain new significant findings to shed light into this highly important area of research to ultimately aid in improving the health of children and adolescents due to the rigorous PRISMA guidelines as well as the high quality peer review standards of PLOS ONE.

7. PLOS authors have the option to publish the peer review history of their article. If published, this will include your full peer review and any attached files.

   If you choose “no”, your identity will remain anonymous but your review may still be made public.

**Do you want your identity to be public for this peer review?** For information about this choice, including consent withdrawal, please see our [Privacy Policy](https://www.plos.org/privacy-policy).

Reviewer #3: No

Answer: Thank you very much for your kind and positive feedback. We were very happy about your positive feedback considering the great efforts we put into this work.
